# Supplementary material for: Predicting regrowth of low-grade gliomas after radiotherapy
Source: PLoS Comput Biol. 2023 Mar 31;19(3):e1011002. doi: 10.1371/journal.pcbi.1011002 (PMC10128962; doi:10.1371/journal.pcbi.1011002)
Supplement: Attachment — Submitted filename: answer_referee.pdf [file pcbi.1011002.s002.pdf]

Answer to referee

We thank the referee for the pertinent remarks. Please find below our comments on the actions that we have taken.

>Section 1.4 appears to be incorrectly formatted. Perhaps this is meant to be a single paragraph?

Yes formatting is poor. This was because the figure+table were within the text. It was changed.

>Pg 6 line 135 "It has no sense to use it on…" is awkwardly phrased
indeed. We rephrased it to "It should not be used it on the previous fits (since it was derived from them), but we checked that ..."

>Pg 7 line 158 "...quite precise, they lie within a few months" a prediction accurate 'within a few months' is arguably not too precise.

Yes in an absolute sense, this is not very accurate. However given the little amount of information and the large spread among patient's reaction to the treatment, this is rather successful. We have rewritten it in this way:
"The tmin predictions for most of the patients lie within a few months of the value determined with all data, which is quite successful given the little amount of information and the fact that each patient react differently to the treatment."

>Figure 6 caption: "This is repeated 1,000 times".... Can the authors provide a specific rationale for this number of monte carlo simulations? Recognizing that this is simply a choice, was the limitation computational time? A sufficient characterization of the distribution? Randomly selected?

Indeed this is an empirical result; we have seen that the results become stable somewhere after a few hundred realizations. We changed the text to
"Since we noticed that a few hundreds of iterations is sufficient to reach a stable distribution, we repeat the procedure 1000 times."

>Figures would benefit from more clear axis labels and legends embedded within the image. For example, axis labels and units for figure 2, meaning of colored lines in figures 6,8

We have reviewed all the figures trying to make them clearer (fig2 and 5 are very crowded so we left the information in the captions)

>Data, but not computational codes are provided

We released the code corresponding to fig6, 7 and 8 which contain all our major methods.
