# Supplementary material for: Predicting regrowth of low-grade gliomas after radiotherapy
Source: PLoS Comput Biol. 2023 Mar 31;19(3):e1011002. doi: 10.1371/journal.pcbi.1011002 (PMC10128962; doi:10.1371/journal.pcbi.1011002)
Supplement: Attachment — Submitted filename: referee3.pdf [file pcbi.1011002.s003.pdf]

Answer to referee

We thank the referee for the review and answered to it in the following way

Reviewer #1: This manuscript deals with an interesting and relevant problem: the prediction of regrowth of low-grade gliomas after radiotherapy. Although the methods used are simple computationally speaking, the result is interesting enough to warrant publication. I recommend it for publication although I think it may benefit from incorporation of several elements into the discussion:

1) Discussing to what extent radiation therapy alone is still used for treatment of low-grade gliomas and how the method could be extended to deal with combinations of radiotherapy and chemotherapies.

Nowadays, treatment for patients with glioma starts as soon as possible just after the diagnosis (and this is also the case for low-grade gliomas). We used this unique dataset because it contains the follow-up of tumors before the beginning of the treatment, that we couldn't not have nowadays. But it is true that irradiation is no longer used alone (and as a treatment of first intention) for treatment of low-grade gliomas. Radiotherapy is still a first intention treatment for high-grade gliomas, but it is clear that we would have far less data in the case of high-grade gliomas (and especially in the part before treatment), since clinicians start the treatment right after the diagnosis. This is why we started with this very nice (but maybe old) dataset.
Now, of course, the same approach could be used to model the response to RT of high-grade gliomas. Actually we would start with the hypothesis that the same model applies to HGG, but the values of the growing velocity before treatment will be much larger.

The other possibility is to continue with low-grade gliomas, but include the effect of chemotherapy alone, or the mixed effects of RT+chemotherapy.
The model would have to be adapted to include the effects of chemotherapy (in this case we cannot make the assumption that the effect of the treatment is only at time t=0), but the approach could be the same. This topic will be our next work and we have adapted the conclusion accordingly.

2) The weakest point of the manuscript in my opinion is the fact that the relationship between tmin and vd is purely empirical. I wonder if using a simplified tumor response to radiation therapy may help in obtaining an explanation for that law, e.g. something such as the calculation of the nadir in Lorenzo et al, J Roy Soc Interf (2019), doi:10.1098/rsif.2019.0195 that describes also response to radiotherapy for a type of slowly-growing, although different, cancer. Indeed that formula is also a time to progression as well as the one

derived for brain metastasis after radiation therapy in Leon-Triana et al, doi:10.3390/math9070716. It may be relevant to discuss the relationship of the approach contained in the manuscript with those explicit formulae.

This correlation, although interesting is not the main results of the paper since it is used only in a few cases (for slow responders). It is indeed only empirical and very non trivial. It does not come from the model itself but really from correlations between the parameters (this is now clarified in the text). The fact that the parameters are correlated certainly means something for the biological processes but what exactly is certainly (very) complicated and out of scope for this phenomenological work. We hope some biologists will suggest some reasons for it. We follow exactly the same approach than in the the 2 cited papers: as us, they do not try to relate what happens before RT to what happen after (for instance citing the 1st work "we do not assume any specific formulation for Rd" and the fraction of damaged cells is a free parameter in the second one).
We have updated the text accordingly and since the Lorenzo et al, J Roy Soc Interf (2019), model is very similar to ours (but for the growing phase) we have added a reference to it.
